# Improved Chloride Tolerance of Pt Surface by Ionomer Layer Structure Engineering

**DOI:** 10.1002/cssc.202402763

**Published:** 2025-04-21

**Authors:** Jongmin Lee, Jongsu Noh, Vy Thuy Nguyen, Chi‐Yeong Ahn, Hyeyoung Shin, Dong Young Chung

**Affiliations:** ^1^ Department of Chemical and Biomolecular Engineering Korea Advanced Institute of Science and Technology Daejeon 34141 Korea; ^2^ Graduate School of Energy Science and Technology (GEST) Chungnam National University Daejeon 34134 Korea; ^3^ Alternative Fuels and Power System Research Center Korea Research Institute of Ships and Ocean Engineering (KRISO) Daejeon 34103 Korea; ^4^ Department of Green Mobility University of Science and Technology (UST) Daejeon 34113 Korea

**Keywords:** activities, chlorides, impurities, local concentrations, stabilities

## Abstract

While recent advancements in electrocatalysts have led to significant progress toward the commercialization of electrochemical energy conversion devices, performance degradation derived by airborne impurity remains a critical challenge to address. In particular, chloride (Cl^−^) poisoning of platinum (Pt) catalysts remains a critical challenge for performance. Herein, an effective strategy for suppressing Cl^−^ poisoning from the perspective of ionomer layer engineering is demonstrated. From the hybrid interface of cation and anion exchange ionomers, the local microenvironment at the catalyst surface is modified, resulting in significant suppression of Cl^−^ poisoning. In situ inductively coupled plasma‐mass spectrometry analysis revealed that the local Cl^−^ concentration at the Pt surface decreased by 40% compared to the bulk concentration. These findings highlight the synergistic role of the hybrid ionomer interface in suppressing Cl^−^ poisoning, validating its effectiveness in maintaining activity and mitigating Pt dissolution. This ionomer engineering approach provides a promising pathway for improving the reliability of electrocatalytic systems under challenging operational conditions.

## Graphical abstract

A hybrid ionomer interface combining cation and anion exchange ionomers effectively suppresses Cl^−^ poisoning of Pt electrocatalysts. In situ inductively coupled plasma‐mass spectrometry measurements reveal a 40% reduction in local Cl^−^ concentration on Pt, attributed to the surface charge distribution of the hybrid ionomer layers. This approach provides a robust strategy for mitigating the effects of anionic impurities in electrocatalysis.

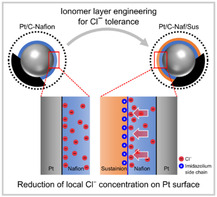

## 1 Introduction

As global awareness of climate change intensifies, the focus on environmentally friendly energy production and utilization has grown significantly.^[^ ^1^, ^2^ ^]^ Hydrogen has emerged as a promising green energy carrier due to its high energy conversion efficiency and clean product (water), making it an attractive alternative to fossil fuels.^[^ ^3^, ^4^ ^]^ Especially, electrochemical reactions for hydrogen production and utilization have gained widespread attention for their efficiency and simplicity of the systems.^[^ ^5^ ^]^ Over the past few decades, the advancements in electrocatalysts for these reactions have enhanced their feasibility. However, as electrochemical energy conversion devices move toward commercialization, the impact of unavoidable impurities during operation has become a critical challenge that must be addressed.^[^ ^6^ ^]^ Impurities in electrochemical systems, including various cations and anions present in water, are known to severely affect catalyst activity, stability, and selectivity.^[^ ^7^, ^8^, ^9^, ^10^, ^11^ ^]^ Among these impurities, Cl^−^ ions are particularly detrimental. They are introduced into the system as byproducts of hydrogen production processes or from airborne contaminants, such as aerosol in marine environments, and pose significant challenges to catalyst performance.^[^ ^12^, ^13^ ^]^

The effects of Cl^−^ poisoning on Pt catalysts can be categorized into two major aspects: (1) the blocking of Pt active sites for oxygen adsorption,^[^ ^14^, ^15^, ^16^, ^17^ ^]^ and (2) the acceleration of Pt dissolution into stable Pt‐chloride complexes, such as PtCl_4_^2−^ and PtCl_6_^2−^.^[^ ^18^, ^19^, ^20^ ^]^ However, the latter one leads to irreversible catalyst degradation, including agglomeration and coarsening through Ostwald ripening, resulting in a severe decrease in the electrochemically active surface area (ECSA) and oxygen reduction reaction (ORR) performance due to increased overpotentials. The effect of Cl^−^ on Pt dissolution has been observed and discussed using the electrochemical quartz crystal microbalance (EQCM) technique.^[^ ^21^, ^22^, ^23^ ^]^ Furthermore, in situ inductively coupled plasma mass spectrometry (ICP‐MS) has been employed for direct monitoring, providing deeper insights into the Pt dissolution mechanism through Pt‐Cl^−^ complex formation.^[^ ^24^, ^25^ ^]^ Not surprisingly, these effects collectively lead to severe performance degradation in proton exchange membrane fuel cells.^[^ ^26^, ^27^, ^28^, ^29^ ^]^

In response to these challenges, various approaches have been explored to improve the Cl^−^ tolerance of Pt‐based catalysts. However, due to the strong poisoning effect of Cl^−^, most studies have primarily focused on removing Cl^−^ impurities from the catalyst layer in fuel cells rather than enhancing the intrinsic Cl^−^ tolerance of the catalysts themselves.^[^ ^27^, ^29^, ^30^, ^31^ ^]^ Recently, Sun et al. reported that PtCo alloy catalysts with a Pt‐skin structure exhibit improved Cl^−^ tolerance due to weakened Pt‐Cl^−^ interactions.^[^ ^32^ ^]^ Additionally, there have been reports on enhancing extrinsic Cl^−^ tolerance by functionalizing the carbon support for Pt nanoparticles, alongside efforts to improve the intrinsic Cl^−^ tolerance of the active material itself.^[^ ^33^ ^]^ However, the role of ionomers in Cl^−^ poisoning of Pt catalysts remains largely unexplored. Since ionomers are directly coated onto the catalyst surface, they can effectively modify the local environment at the catalyst‐electrolyte interface.^[^ ^34^, ^35^ ^]^ Therefore, engineering the ionomer layer structure has the potential to create unique interfacial characteristics that are not commonly observed.

In this study, we demonstrate that engineering ionomer layer structures provides a straightforward solution to enhance the Cl^−^ tolerance of Pt ORR catalysis. From the hybrid structure of cation exchange ionomer (Nafion) and anion exchange ionomer (Sustainion) layers, we report their synergistic effects in reducing local Cl^−^ concentrations near the Pt surface, thereby enhancing ORR activity and suppressing Pt dissolution under potential cycling conditions. Using in situ ICP‐MS analysis, we quantitatively measured Pt dissolution as a function of Cl^−^ concentrations, revealing the impact of these ionomer configurations on the local environment. The reduction in local Cl^−^ concentration within the bilayer ionomer structure originates from electrostatic interactions induced by the charge distribution of the ionomer side chains.

## 2 Results and Discussion

### 2.1 The Effect of the Ionomer Structure Engineering on Cl^−^

**Figure 1:**
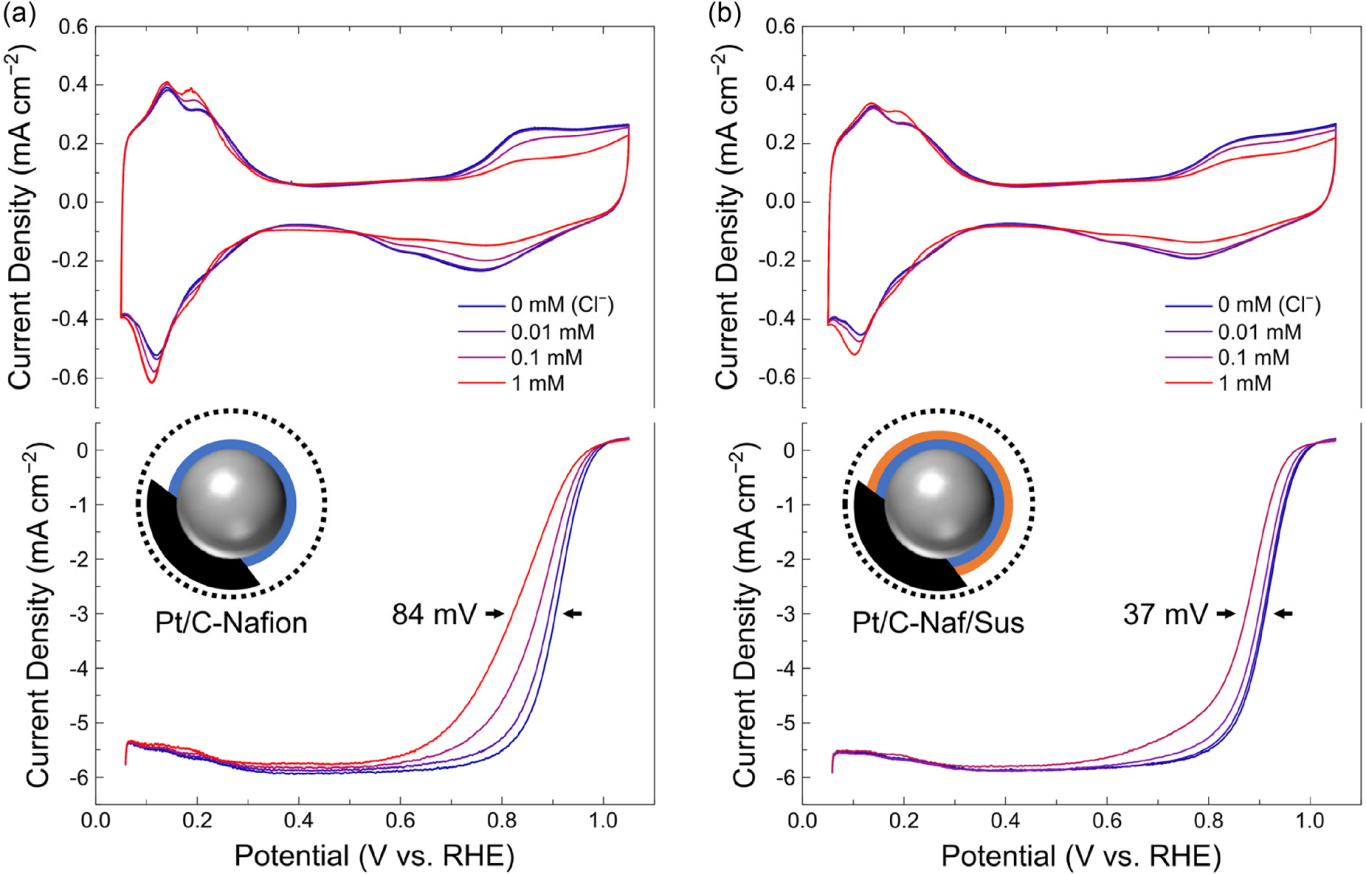
Cyclic voltammetry (CV) and oxygen reduction reaction (ORR) curves of ionomer coating‐engineered Pt/C catalysts in 0.1 M HClO_4_ containing chloride (Cl^−^) ion impurities (0, 0.01, 0.1, and 1 mM). a) Nafion‐coated, b) Naf/Sus‐coated Pt/C.

To investigate the influence of ionomer structures on the Cl^−^ resistance of Pt surfaces, two types of Pt/C electrodes were prepared: one coated solely with Nafion ionomer and the other hybrid coated with both Nafion and Sustainion ionomers (Naf/Sus). Cyclic voltammetry (CV) and ORR measurements were conducted with incremental additions of NaCl, increasing Cl^−^ concentrations by order of magnitude from 0.01 to 1 mM (**Figure** **1**a,b). As reported in previous studies, increasing Cl^−^ concentrations led to a slight increase in the current within the *H*_upd_ region due to Cl^−^ adsorption/desorption and a decrease in the OH adsorption region due to Cl^−^ poisoning.^[^ ^17^, ^19^, ^23^, ^32^ ^]^ This trend was evident as ORR performance gradually decreased with increasing Cl^−^ concentrations. For the Nafion‐coated electrode, the half‐wave potential decreased by 84 mV at 1 mM Cl^−^ compared to a Cl^−^‐free electrolyte. In contrast, the Naf/Sus‐coated electrode exhibited a smaller decrease of 37 mV, demonstrating enhanced resistance to Cl^−^ poisoning. These findings indicate that the introduction of a Sustainion ionomer layer on top of Nafion significantly improves the Cl^−^ resistance of the Pt surface. To elucidate the role of Sustainion, an electrode coated solely with Sustainion was also tested (Figure S1, Supporting Information). Unlike Nafion or Naf/Sus structures, the Sustainion‐coated electrode displayed a dramatic reduction in OH adsorption current and a substantial 160 mV drop in the ORR half‐wave potential at 1 mM Cl^−^. Moreover, control experiments employing a reversed ionomer configuration (Sustainion/Nafion) exhibited a markedly lower limiting current and greater Cl^−^ sensitivity, supporting that the ionomer layers remain spatially distinct and that the configuration of the ionomer layers is a key factor governing Cl^−^ mitigation (Figure S2, Supporting Information).

Despite the differences in Cl^−^ tolerance among the ionomer layer structures, all three electrodes exhibited identical ORR performance in Cl^−^‐free 0.1 M HClO_4_ electrolyte (Figure S3, Supporting Information). This confirms that the inherent electrochemical activity of the Pt catalysts was not affected by the ionomer structure. However, CV curves revealed a trend of decreasing ECSA, calculated from the *H*_upd_ region, in the order of Nafion > Naf/Sus > Sustainion (Table S1, Supporting Information). The reduction in ECSA is attributed to the site‐blocking effect of the imidazolium structure in Sustainion, which strongly adsorbs onto the Pt surface, as reported previously.^[^ ^36^, ^37^ ^]^ Despite a reduction in the *H*_upd_ current, the ORR performance remained largely unaffected. This can be attributed to the preferential blocking of low‐coordinated sites, which are less active for ORR, while highly active terrace sites (e.g., Pt(111)) remain accessible.^[^ ^36^ ^]^ Furthermore, differences in the electrochemical environment between the *H*_upd_ and ORR regions, including potential‐dependent adsorption behavior and electrical double layer effects, can decouple the apparent ECSA from actual ORR activity.^[^ ^38^, ^39^, ^40^, ^41^, ^42^, ^43^ ^]^ Accordingly, Nafion and Sustainion hybrid ionomer systems effectively enhance the Cl^−^ resistance of Pt surfaces without compromising ORR activity.

### 2.2 Pt Dissolution Study Correlated with Cl^−^ Tolerance Behavior

**Figure 2:**
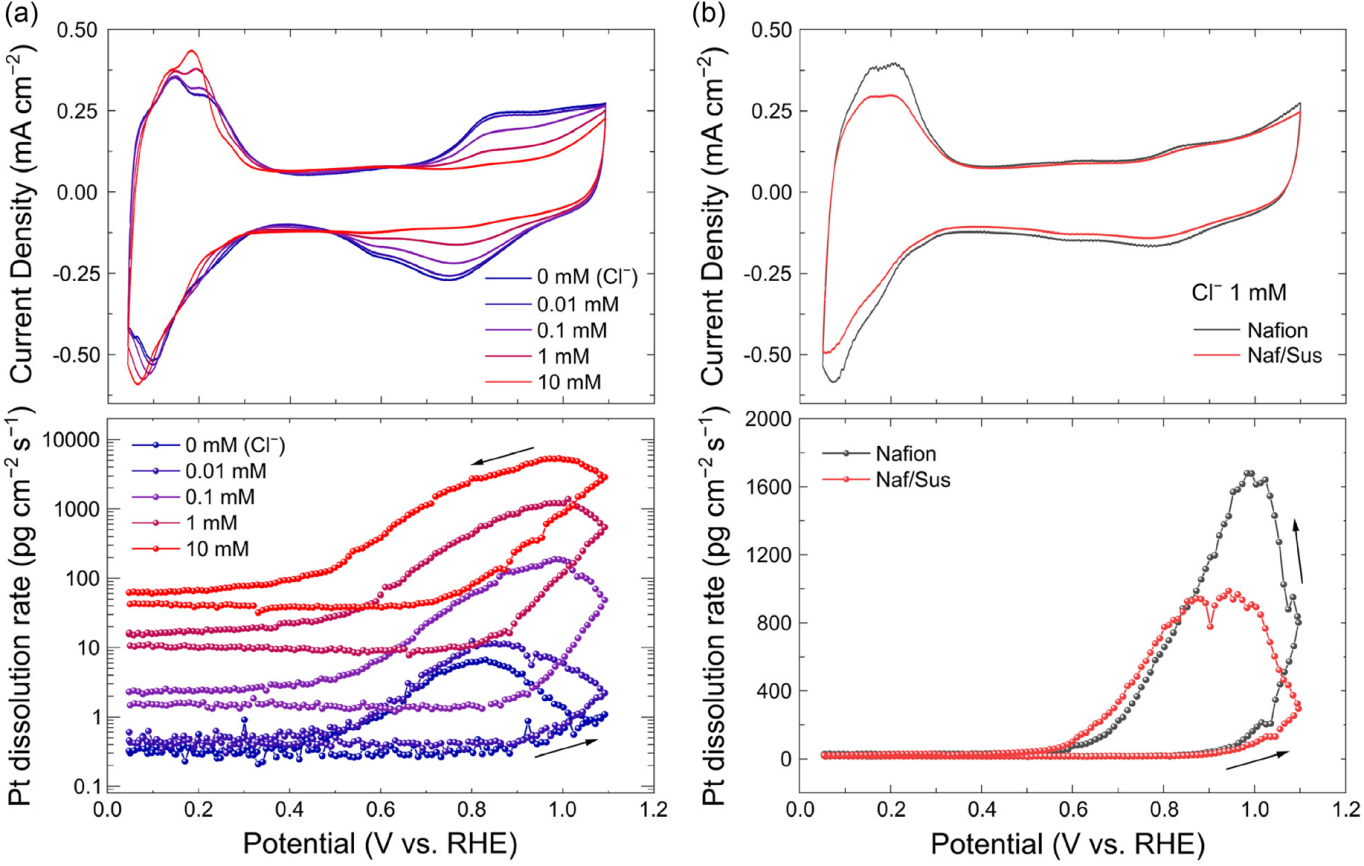
Simultaneous in situ evaluation of CV curves (top) and potential‐resolved Pt dissolution rate through ICP‐MS (bottom). a) Nafion‐coated Pt/C in various concentrations of Cl^−^. b) Nafion and Naf/Sus‐coated Pt/C at 1 mM of Cl^−^.

**Figure 3:**
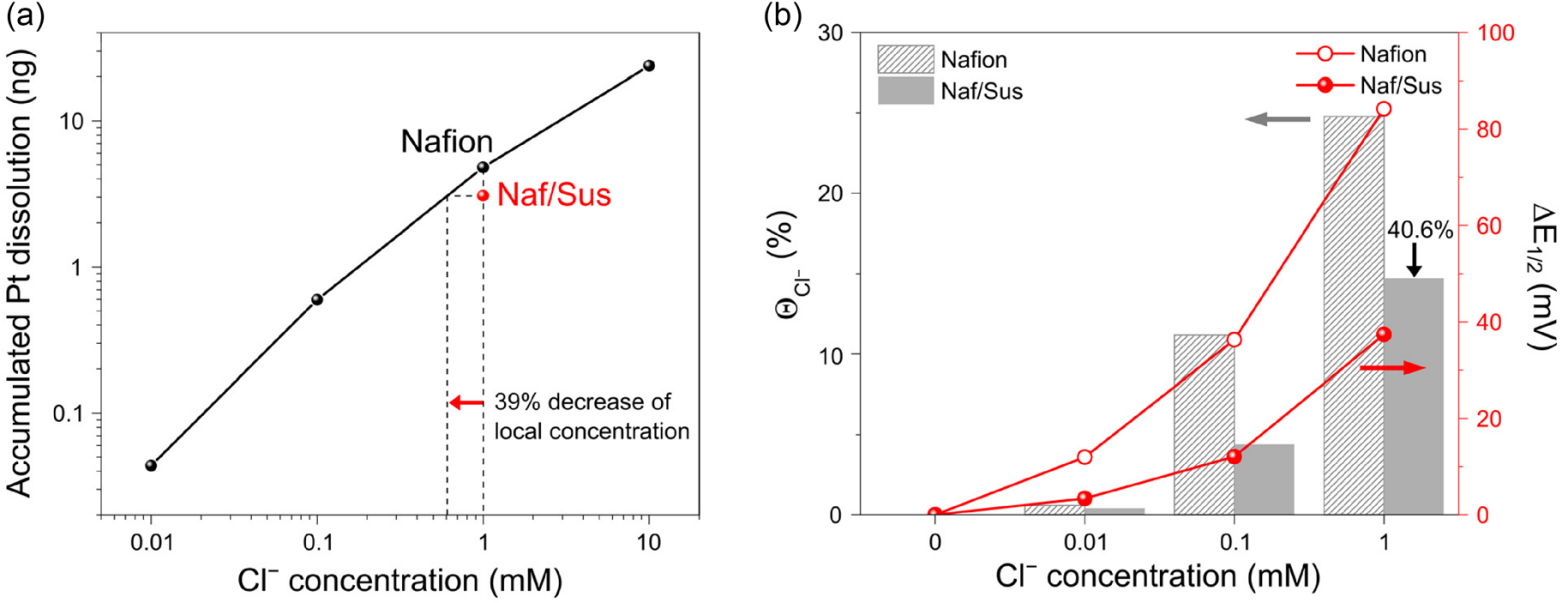
Determination of the local concentration of Cl^−^. a) Estimation from the accumulated Pt dissolution calculated by in situ ICP‐MS measurement and b) ORR half‐wave potential (line) and Cl^−^ coverage (bar) calculated from the CV curves up to 0.9 V.

Given that the presence of Cl^−^ negatively impacts both activity and stability, investigating dissolution behavior provides a direct method to estimate the degree of interaction between Pt and Cl^−^ anions at the electrode/electrolyte interface.^[^ ^23^ ^]^ Before examining the effect of ionomer structure on Pt dissolution, we first verified the Pt dissolution behavior of a Nafion‐coated electrode under Cl^−^ poisoning conditions by in situ ICP‐MS (**Figure** **2**a). Without Cl^−^ ions, Pt dissolution began during the anodic scan but became more prominent during the cathodic scan, corresponding to transient dissolution associated with Pt‐oxide reduction or OH desorption. However, the introduction of Cl^−^, with concentrations increasing by an order of magnitude, significantly increases the Pt dissolution rate, which is well aligned with previous results.^[^ ^24^, ^25^ ^]^ To investigate the effect of ionomer structures on Pt dissolution, we measured the Pt dissolution rate in an electrolyte containing 1 mM of Cl^−^ (Figure 2b). Notably, the Naf/Sus‐coated electrode exhibited a significantly lower Pt dissolution rate compared to the Nafion‐coated electrode (note: Pt dissolution rate was slightly higher for the Naf/Sus‐coated electrode in Cl^−^‐free electrolyte as shown in Figure S4, Supporting Information). Therefore, the decrease of Pt dissolution rate originated from the attenuated interaction between Pt and Cl^−^ by engineered ionomer interface, consistent with the enhanced Cl^−^ poisoning resistance observed in ORR measurements. Based on the fact that a linear correlation between the Cl^−^ concentration and the accumulated dissolution of Pt was observed in **Figure** **3**a and Table S2, Supporting Information, the amount of Pt dissolution can serve as a quantitative indicator of the local concentration of Cl^−^ on Pt. This linear correlation was also observed in previous reports utilizing EQCM and ICP‐MS techniques that monitored Pt dissolution in Cl^−^ (Figure S5, Supporting Information).^[^ ^23^, ^24^ ^]^ From this correlation, we investigated the local concentration of Cl^−^ in the Naf/Sus‐coated electrode. The estimated local Cl^−^ concentration was ≈0.61 mM, corresponding to 61% of the bulk concentration of 1 mM. This suggests that the Naf/Sus ionomer interface effectively reduces the local Cl^−^ concentration at the Pt‐ionomer interface by 39%.

To further validate the reduction in local Cl^−^ concentration on the Pt surface, Cl^−^ coverage (*θ*_Cl_^−^) analysis was conducted (Figure 3b and Table S3, Supporting Information). The *θ*_Cl_^−^ was determined based on the OH adsorption charge up to 0.9 *V*_RHE_ to exclude interference from Pt‐oxide formation. It is assumed that the total *H*_upd_ charge on the Nafion‐coated electrode in Cl^−^‐free electrolyte corresponds to 1 monolayer (ML) coverage of Pt surface.^[^ ^44^ ^]^ From this assumption, the OH adsorption (OH_ad_) charge was normalized to calculate the OH coverage (*θ*_OH_)

(1) $$\theta_{\text{OH}}=\frac{Q_{\text{OH}}}{Q_{H\text{upd}}}$$

*θ*_Cl_^−^ was then defined as the reduction in *θ*_OH_ at each Cl^−^ concentration compared to the Cl^−^‐free electrolyte^[^ ^45^ ^]^

(2) $$\theta_{{\text{Cl}}^{-}}=\;\theta_{{\text{OH, Cl}}^{-}\text{‐free}}-\theta_{{\text{OH, Cl}}^{-}}$$

At the Cl^−^ concentration of 1 mM, the *θ*_Cl_^−^ was 0.25 ML for the Nafion‐coated electrode and 0.15 ML for the Naf/Sus‐coated electrode, indicating a 60.4% reduction. This result confirms that the reduction in local Cl^−^ concentration calculated from in situ Pt dissolution measurements directly correlates with the *θ*_Cl_^−^ reduction observed in the CV analysis. This correlation provides a validation of the local Cl^−^ concentration reduction effect achieved by the engineered ionomer structure at the electrode surface. It is also well‐fitted to the degradation trend of half‐wave potential in ORR, indicating the *θ*_Cl_^−^ is the key descriptor for elucidating the performance in the presence of Cl^−^.

**Figure 4:**
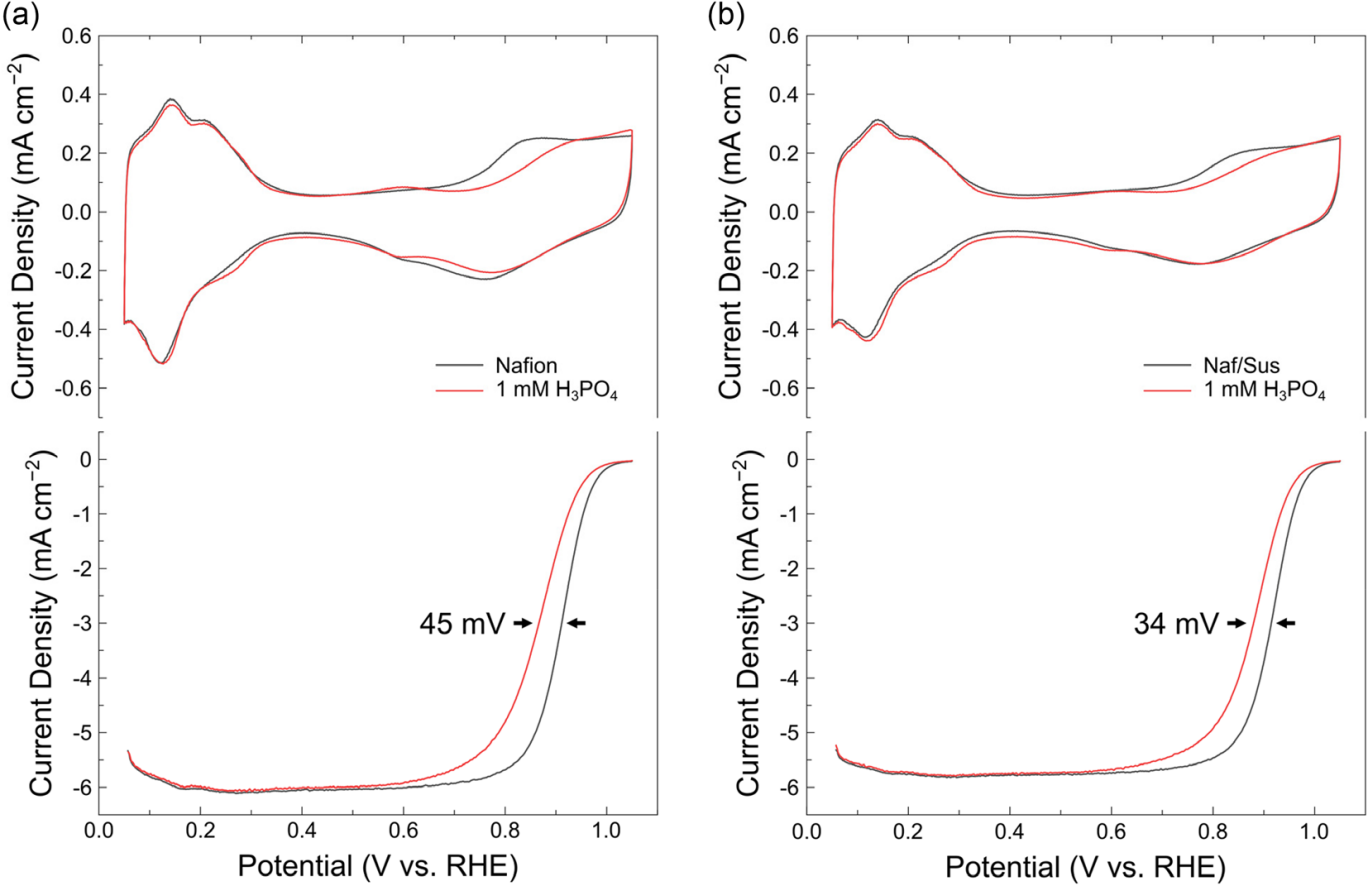
CV and ORR curves of a) Nafion‐coated and b) Naf/Sus‐coated Pt/C in pure electrolyte and the electrolyte containing 1 mM of H_3_PO_4_.

To evaluate whether the local anion concentration reduction effect observed with the Naf/Sus‐coated electrode extends to other anions, we conducted electrochemical measurements before and after poisoning with phosphate (PO_4_^3−^) anion, known for its strong poisoning effect on Pt surfaces (**Figure** **4**a,b).^[^ ^46^ ^]^ After the phosphate ion was introduced, there was a peak observed near 0.6 V in CV curves which corresponds to phosphate adsorption/desorption on the Pt surface.^[^ ^47^ ^]^ The charge density of this peak was attenuated for the Naf/Sus‐coated electrode compared to the Nafion‐coated electrode. Furthermore, the ORR half‐wave potential loss was less pronounced for the Naf/Sus‐coated electrode, confirming that this ionomer interface mitigates anion poisoning effects not only for Cl^−^ but also for other anions such as PO_4_^3−^. Therefore, it can be inferred that the anti‐poisoning behavior of the Naf/Sus ionomer structure is attributed to the surface charge characteristics of the ionomer layers.

### 2.3 Origin of the Local Cl^−^ Reduction in Bilayer Ionomer Structure

**Figure 5:**
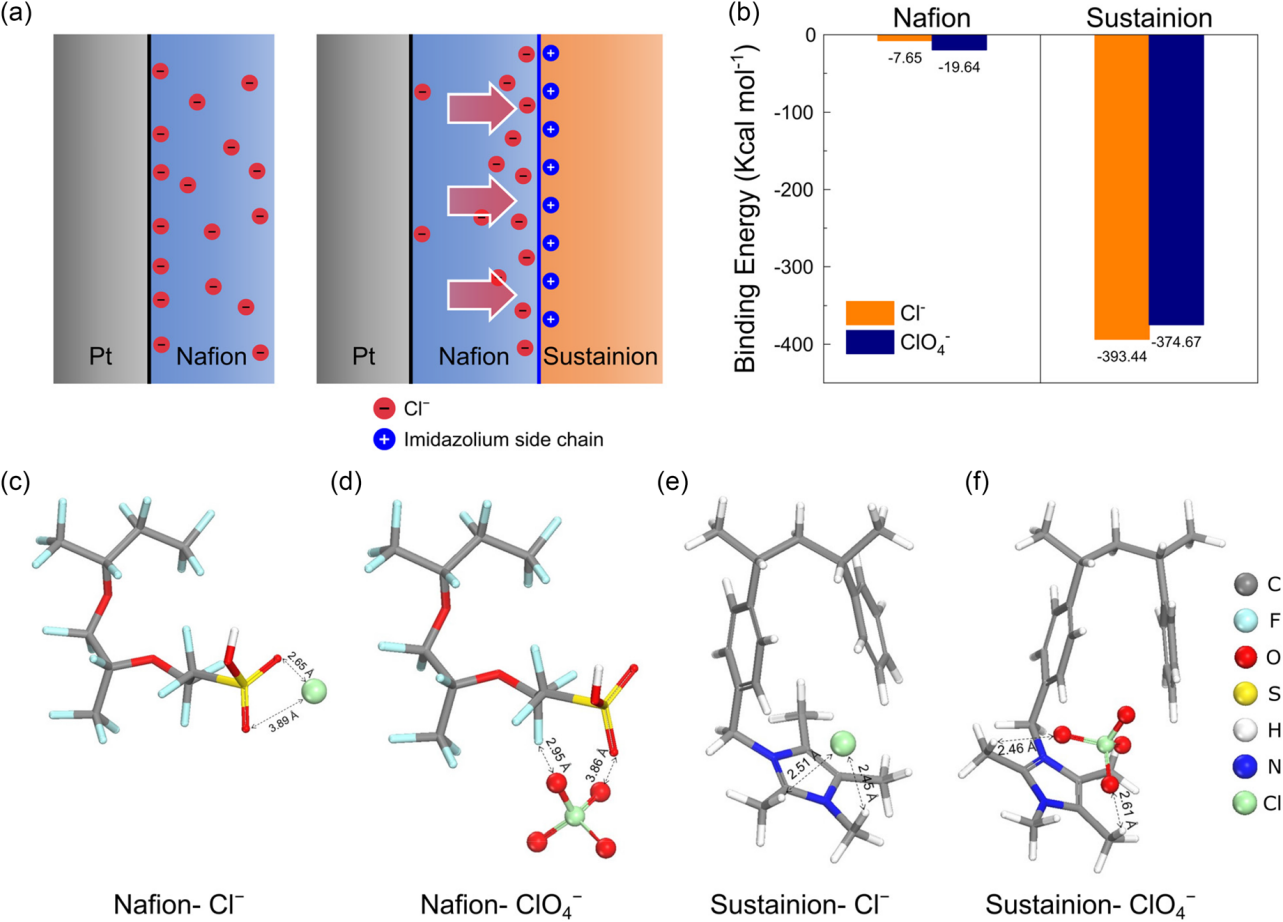
Schematic diagram of the Cl^−^ behavior in the ionomer structure and supporting DFT calculation results. a) The schematic distribution profile of Cl^−^ ion at the catalyst‐ionomer interface of Pt/C‐Nafion electrode and Pt/C‐Naf/Sus hybrid electrode. b) The corresponding binding energies between anions (Cl^−^/ClO_4_^−^) and protonated Nafion and *cis*‐Sustainion C‐ring bound. Optimized structures of c,d) protonated Nafion and e,f) *cis*‐Sustainion C‐ring bound to the respective anions (Cl^−^/ClO_4_^−^). Gray, cyan, red, yellow, white, blue, and green spheres correspond to C, F, O, S, H, N, and Cl atoms, respectively.

To elucidate the mechanism by which the hybrid Naf/Sus structure inhibits Cl^−^ adsorption on Pt, we propose a surface charge‐induced electrostatic interaction model, as illustrated in **Figure** **5**a. Based on the chemical structures of Nafion and Sustainion, their respective roles in influencing Cl^−^ behavior at the Pt surface can be analyzed. Nafion, a cation‐exchange ionomer, contains sulfonyl group side chains that confer a negative background charge. In contrast, Sustainion, an anion‐exchange ionomer, features imidazolium group side chains with a positive charge due to quaternary ammonium groups.^[^ ^35^ ^]^ The charge distributions of these ionomers enable their synergistic roles in mitigating Cl^−^ poisoning when used in a bilayer configuration. The Nafion layer, positioned directly adjacent to the Pt surface, induces a Donnan exclusion effect that restricts anion transport and reduces Cl^−^ permeability to the catalyst surface.^[^ ^48^ ^]^ This exclusion results in a lower local concentration of Cl^−^ at the Pt‐Nafion interface. When a Sustainion layer is introduced on top of the Nafion layer, the positive charge of Sustainion attracts Cl^−^ ions toward the outer ionomer layer, effectively repelling them from the Pt‐Nafion interface. This layered configuration further reduces the local Cl^−^ concentration at the catalyst surface. To validate these interactions at the atomic and molecular levels, density functional theory (DFT) calculations were conducted. As shown in the binding energy results (Figure 5b) and molecular geometries (Figure 5c–f), the binding energy between Cl^−^ and the sulfonate groups of Nafion is relatively weak, assuming full protonation of the sulfonate groups under low pH conditions. Notably, ClO_4_^−^ exhibited stronger interactions with Nafion compared to Cl^−^, supporting the hypothesis that Cl^−^ ions are preferentially excluded from the Nafion layer. In Sustainion, however, Cl^−^ exhibited the strongest interaction compared to ClO_4_^−^ and OH^−^ (Figure S6e, Supporting Information). These results suggest that Cl^−^ ions excluded by Nafion are captured at the Nafion/Sustainion interface, creating a local deficiency of Cl^−^ at the Pt/Nafion interface when Sustainion is introduced. We further investigated the Cl^−^ tolerance behavior at higher concentrations, demonstrating the mitigation effect remained effective up to 1 mM Cl^−^ but was significantly reduced at 10 mM. This indicates the presence of a saturation threshold of imidazolium groups under Cl^−^ exposure. Considering that practical Cl^−^ contamination levels in PEMFCs (e.g., seawater aerosol) are several orders of magnitude lower (0.001–0.01 ppm), the Nafion/Sustainion bilayer is expected to offer robust protection under realistic operating conditions (Figure S7, Supporting Information).^[^ ^49^ ^]^ This finding highlights the critical importance of designing effective ionomer layer architectures to minimize Cl^−^ poisoning and improve catalyst durability.

## 3 Conclusion

In summary, we have demonstrated that engineering ionomer layer structures can effectively enhance the Cl^−^ tolerance of Pt catalysts, a critical factor for performance degradation in electrocatalysis. Through the hybrid system of Nafion and Sustainion ionomers on electrodes, we achieved enhanced Cl^−^ tolerance. Using in situ ICP‐MS, we quantified the local concentration of Cl^−^ on the Pt interface. The hybrid ionomer interface structure significantly reduced the local Cl^−^ concentration near the Pt surface, thereby enhancing ORR activity and suppressing Pt dissolution during potential cycling. This reduction in local Cl^−^ concentration is attributed to electrostatic interactions arising from the charge distribution of the ionomer side chains. Our findings highlight the critical role of ionomer layer structure engineering in electrode design strategies for mitigating the effects of anionic impurities in electrochemical systems.

## 4 Experimental Section

4.1

4.1.1

##### Materials

Commercial 20 wt% Pt/C (HiSPEC 3000, Johnson Matthey Co.), Nafion ionomer solution (D521, 5 wt%, Dupont), and Sustainion ionomer solution (XA‐9, 5 wt%, Dioxide Materials) were purchased from each distributor. Isopropanol (IPA, 99.9%), hydrochloric acid (HCl, 37%), and perchloric acid (HClO_4_, 70%) were purchased from Sigma‐Aldrich. Sodium chloride (NaCl, 99.999%) was purchased from Alfa‐Aesar. Ultrapure deionized (DI) water (Milli‐Q, 18.2 MΩ, total organic carbon ≤2 ppb) was used for the preparation of catalyst inks and electrolytes.

##### Electrochemical Measurements

The electrochemical measurements were performed using a rotating‐disk electrode (RDE) setup with a modulated speed rotator (PINE, AFMSRCE) and a potentiostat (Autolab, PGSTAT 302 N). A homemade fluorinated ethylene propylene (FEP) cell was cleaned by acid treatment and three times of boiling in DI water to remove trace impurities before the measurements. A glassy carbon (GC) electrode (6 mm diameter, disk area: 0.283 cm^2^), a double‐junction Ag/AgCl (3 M KCl, EC‐Frontier), and a graphite carbon rod (Alfa‐Aesar) were used as working, reference, and counter electrodes, respectively. The catalyst inks were prepared by dispersing Pt/C (5 mg) in DI water and IPA (4 mg mL^−1^) with Nafion or Sustainion ionomer (30 μL) by ultrasonication. Well‐dispersed catalyst inks were drop‐casted on the GC disk and dried with the rotation at 300 rpm for uniformity of the films. For hybrid coating of Nafion and Sustainion, the catalyst film was first prepared by Nafion‐containing ink and then subsequently coated with Sustainion by drop‐casting on the dried film of Nafion‐coated catalyst. The amount of ionomer was controlled the same. The electrode was preconditioned by sequentially rotating in 0.1 M KOH and DI for 10 min each, to fully convert the chloride form of Sustainion to the hydroxide form. The normalized Pt loading was controlled to 20 μg_Pt_ cm^−2^ for all the measurements. All potentials were presented after being referenced to a reversible hydrogen electrode (RHE).

The electrochemical measurements were conducted in 0.1 M HClO_4_ at room temperature. For catalyst activation, the catalysts were pretreated in Ar‐saturated electrolyte in the potential between 0.05 and 1.05 *V*_RHE_ at a scan rate of 50 mV s^−1^ for 30 cycles. Then, the CV curves were obtained in the potential between 0.05 and 1.05 *V*_RHE_ at a scan rate of 20 mV s^−1^. For ORR measurements, the pretreated catalysts were transferred to a fresh O_2_‐saturated electrolyte and cycled in the potential between 0.05 and 1.05 *V*_RHE_ at a scan rate of 20 mV s^−1^ with a rotating speed of 1600 rpm. The iR compensation was applied during ORR measurements with its solution resistance value. To control the Cl^−^ concentration, a specific amount of 3 M NaCl stock solution was spiked into the electrolyte. To minimize the kinetics of Cl^−^ poisoning, ORR measurements were conducted right after the immersion of the electrode.

##### In Situ ICP‐MS Measurements

To measure Pt dissolution behavior, Pt ion (195 amu) was detected using an ICP‐MS (Nexion 2000, PerkinElmer) coupled with an RDE equipped with a stationary probe. The electrochemical cell was configured as a standard three‐electrode system. The working and reference electrodes were prepared consistently with the previous electrochemical measurements, and the counter electrode was a Pt wire (Premion, 99.997%). To minimize interference from the dissolution of the counter electrode, an H‐type cell configuration was employed, consisting of two glass compartments separated by a proton‐exchange membrane (Aquivion E98‐09S, Solvay). Before each measurement, the glass compartments were thoroughly cleaned by boiling in DI water and rinsing several times with fresh DI water.

Before dissolution measurements, each electrode was preconditioned by cycling between 0.05 and 1.1 *V*_RHE_ at a scan rate of 50 mV s^−1^ for 30 cycles in Ar‐saturated 0.1 M HClO_4_ electrolyte. This preconditioning process activated the catalyst and stabilized Pt dissolution. Subsequently, the electrode was transferred to Ar‐saturated fresh electrolyte to obtain accurate measurements of Pt dissolution. The Pt dissolution was then measured under Ar‐saturated conditions during cycling between 0.05 *V*_RHE_ and 1.1 *V*_RHE_ at a scan rate of 20 mV s^−^^1^, with the electrode rotating at 100 rpm. The concentration of Cl^−^ was adjusted by the addition of 0.1 M HCl to minimize the pH change of the electrolyte and cation matrix effect in ICP measurements. The obtained Pt signal was converted to a dissolution rate (unit: pg cm^−2^ s^−1^) using a flow rate of 7.5 μL s^−1^, a geometric area of 0.283 cm^2^, and a collection efficiency of 33%. Accumulated Pt dissolution (unit: ng) was calculated by integrating the area under ICP response (dissolution rate) versus time curves.

##### Computational Details

To investigate the effects of ionomer coating engineering on mitigating Cl^−^/ClO_4_^−^ poisoning on the Pt surface, DFT calculations on binding complexes of both anions and ionomers were carried out using Orca^[^ ^50^ ^]^ v.6.0. The hybrid functional B3LYP^[^ ^51^, ^52^ ^]^ was employed with semiempirical D3 dispersion correction^[^ ^53^ ^]^ using Becke–Johnson^[^ ^54^ ^]^ damping. The def2‐TZVP^[^ ^55^ ^]^ basis set was utilized for all calculations to ensure precise treatment of polarization and dispersion effects of species such as Cl^−^ and ClO_4_^−^ anions. For Nafion and Sustainion structures, geometry optimization and frequency calculations were performed in the gas phase with no symmetry constraints, effectively representing the structural and electronic properties through their respective monomer units. The optimized configurations of both Nafion and Sustainion are illustrated in Figure S6a–c, Supporting Information. After examining both *cis* and *trans* conformations of Sustainion with OH^−^, the *cis*‐Sustainion configuration, in which OH^−^ binds to the C‐ring of imidazolium, has been identified as the most energetically favorable (Figure S6d, Supporting Information) and will thus be employed in subsequent investigations. The binding energies (*E*_bind_) of the anion‐immobilizer complexes were calculated using the following equation

(3) $$E_{\text{bind}}=E_{\text{complex}}\;-\;(E_{\text{anion}}+E_{\text{ionomer}})$$

where *E*_complex_ was the total energy of anion‐ionomer complexes, and *E*_anion_ and *E*_ionomer_ were the energy of isolated anions and ionomers.

## Conflict of Interest

The authors declare no conflict of interest.

## Supporting information

Supplementary Material

## Data Availability

The data that support the findings of this study are available from the corresponding author upon reasonable request.
