# Supplementary material for: Improved Chloride Tolerance of Pt Surface by Ionomer Layer Structure Engineering
Source: ChemSusChem. 2025 Apr 21;18(13):e202402763. doi: 10.1002/cssc.202402763 (PMC12231957; doi:10.1002/cssc.202402763)
Supplement: Supplementary file 1 — Supplementary Material [file CSSC-18-e202402763-s001.pdf]

ChemSusChem
Supporting Information

Improved Chloride Tolerance of Pt surface by Ionomer Layer Structure Engineering

Jongmin Lee,[a]† Jongsu Noh,[a]† Vy Thuy Nguyen,[b]† Chi-Yeong Ahn,[c,d]* Hyeyoung Shin,[b]* Dong Young Chung [a]*

WILEY-VCH

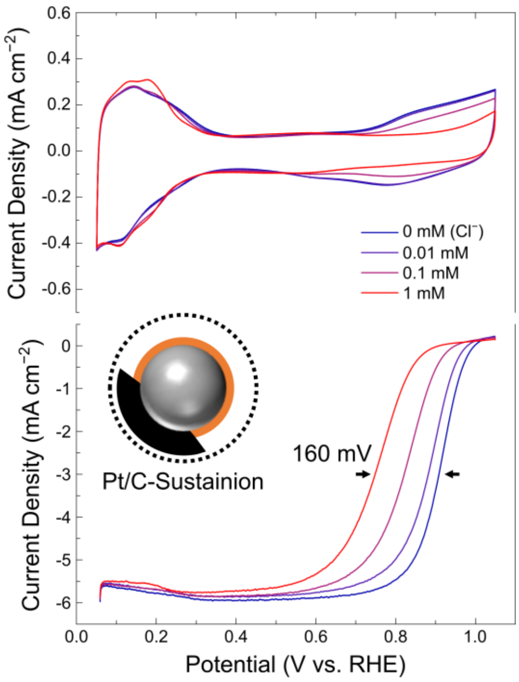

Figure S1. Cyclic voltammetry (CV) and oxygen reduction reaction (ORR) curves of Sustainion-coated Pt/C catalysts in 0.1 M $HClO_4$ containing chloride (Cl^-) ion impurities (0, 0.01, 0.1, and 1 mM).

WILEY-VCH

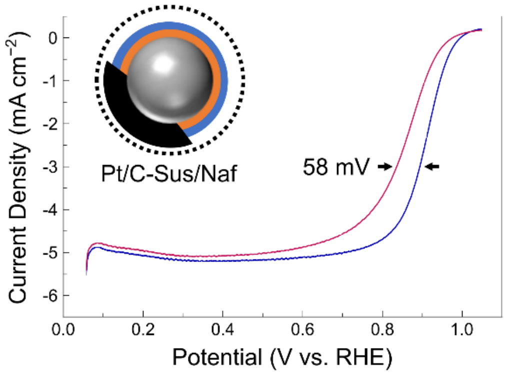

Figure S2. ORR curves of Sus/Naf-coated Pt/C catalysts in 0.1 M $HClO_4$ containing with chloride (Cl^-) ion impurities (0, 1 mM).

WILEY-VCH

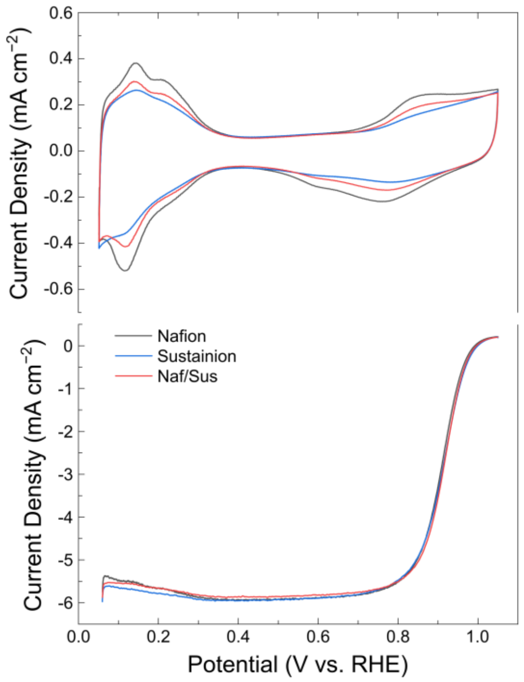

Figure S3. CV (top) and ORR (bottom) curves of Nafion, Sustainion, Naf/Sus-coated Pt/Cs in Cl^{-}-free 0.1 M $HClO_4$.

WILEY-VCH

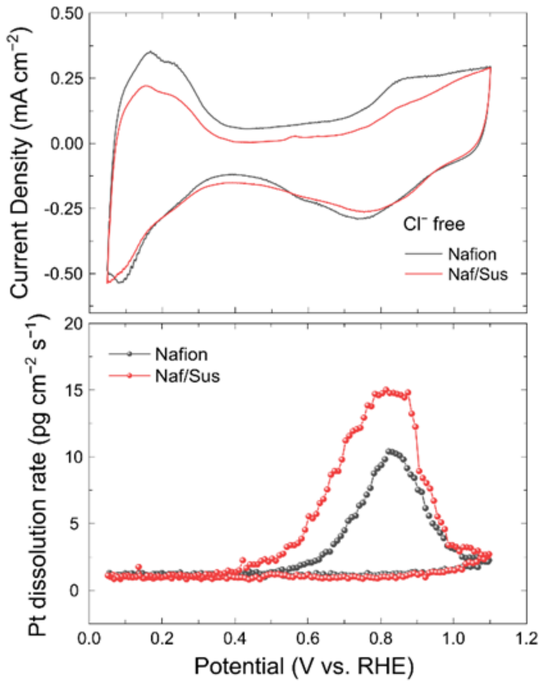

Figure S4. Simultaneous in situ evaluation of CV curves (top) and potential-resolved Pt dissolution through ICP-MS (bottom) of Nafion and Naf/Sus-coated Pt/Cs in Cl^- free electrolyte.

WILEY-VCH

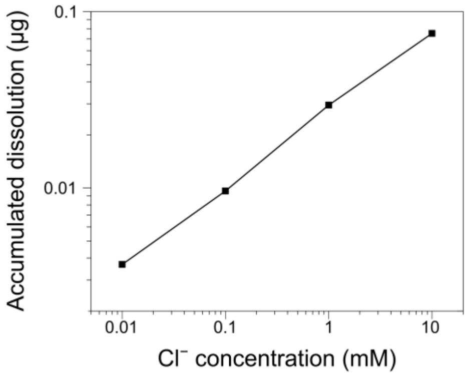

Figure S5. Plot of a logarithmic scale amount of accumulated dissolution as a function of Cl^{-} concentration a logarithmic scale. The plot is generated based on the Pt dissolution measurement data from the reference. Adapted with permission from Ref [24]. Copyright 2014 Royal Society of Chemistry.

WILEY-VCH

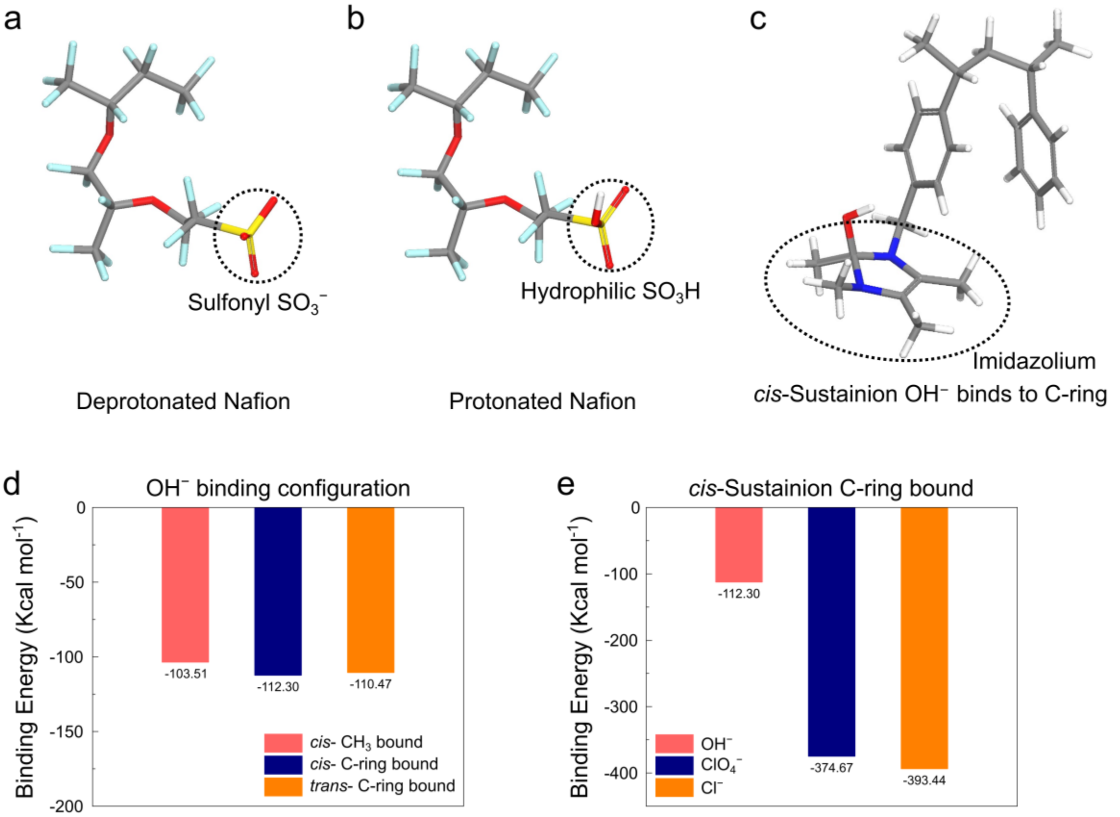

Figure S6. Optimized structural representation of (a) Deprotonated Nafion, (b) Protonated Nafion, (c) The composition of *cis*-Sustainion with Hydroxide ion (OH^-) binding to C-ring of Imidazolium group . Gray, cyan, red, yellow, white, and blue spheres correspond to C, F, O, S, H and N atoms, respectively, (d) Comparison binding energy of OH^- in various Sustainion configurations, (e) The corresponding binding energies between anions (OH^- /ClO_4^-/ Cl^-) and *cis*-Sustainion C-ring bound.

WILEY

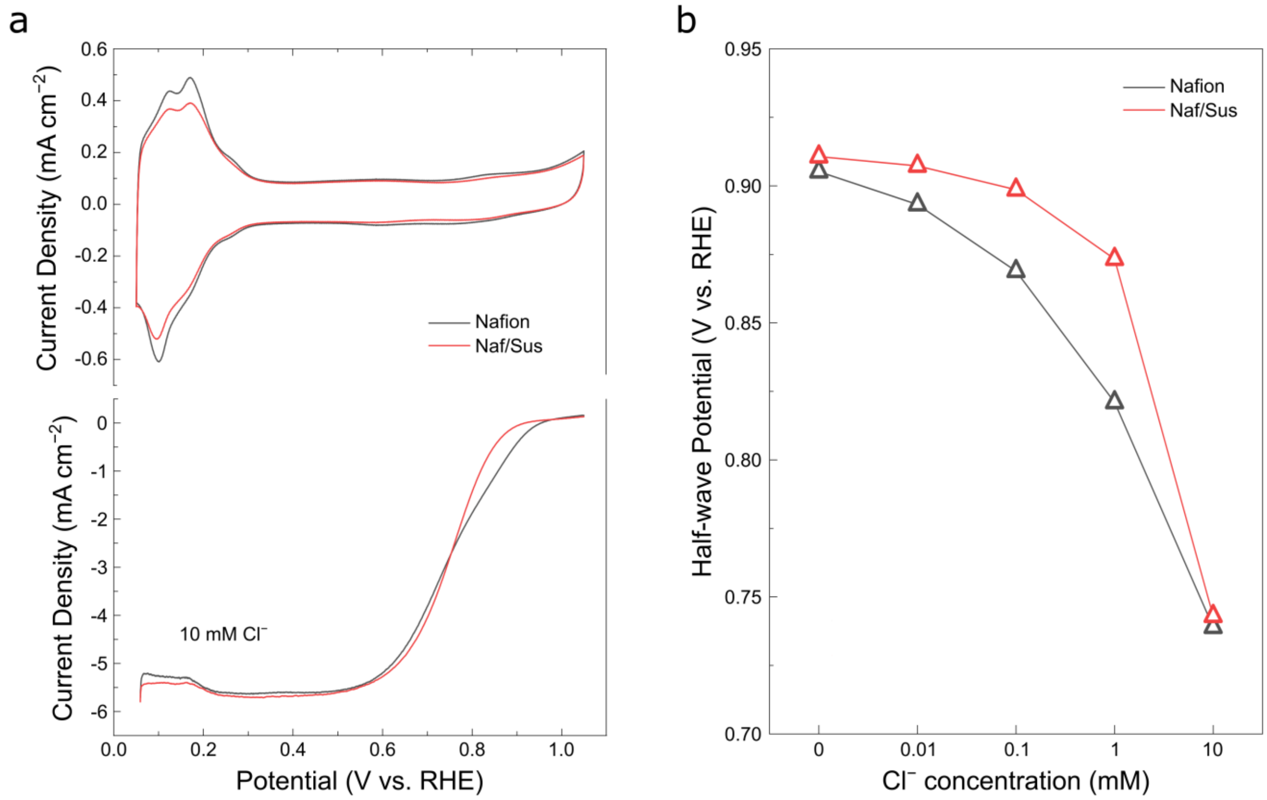

Figure S7. Cl^- saturation behavior. (a) CV and ORR curves in the electrolyte containing 10 mM of Cl^-, and (b) ORR half-wave potential change in various concentration of Cl^- of Nafion-coated and Naf/Sus-coated Pt/C.

WILEY

Table S1. Electrochemically active surface area (ECSA) of ionomer layer structure engineered electrodes calculated from H_{upd} charge in CV curves.

Ionomer layer Structure	Nafion	Naf/Sus	Sustainion
ECSA (m^2/g_{Pt})	69.1	53.38	45.69

Table S2. Acculmulated Pt dissolution (unit: ng) of Nafion and Naf/Sus-coated Pt/Cs at various Cl^- concentrations.

Ionomer layer Structure	Cl^- concentration (mM)			
	**0.01**	**0.1**	**1**	**10**
Nafion	0.044	0.597	4.803	23.761
Naf/Sus	-	-	3.073	-

Table S3. Cl^- coverage(θ_{Cl^-}, unit: %) on Pt surface calculated from OH coverage(θ_{OH}) derived from each CV curves.

Ionomer layer Structure	Cl^- concentration (mM)			
	**0**	**0.01**	**0.1**	**1**
Nafion	0	0.596	11.181	24.779
Naf/Sus	0	0.408	4.391	14.721

WILEY
